# Polymorphisms in xenobiotic metabolizing genes (*EPHX1, NQO1* and *PON1*) in lymphoma susceptibility: a case control study

**DOI:** 10.1186/1471-2407-13-228

**Published:** 2013-05-07

**Authors:** Pablo Conesa-Zamora, Javier Ruiz-Cosano, Daniel Torres-Moreno, Ignacio Español, María D Gutiérrez-Meca, Javier Trujillo-Santos, Elena Pérez-Ceballos, Rocío González-Conejero, Javier Corral, Vicente Vicente, Miguel Pérez-Guillermo

**Affiliations:** 1 Molecular Pathology and Pharmacogenetic Group. Pathology Department, Santa Lucía General University Hospital (HGUSL), 30202, Cartagena, Spain; 2 Haematology Department. (HGUSL), 30202, Cartagena, Spain; 3 Internal Medicine Department. (HGUSL), 30202, Cartagena, Spain; 4 Haematology Department, Morales Meseguer University Hospital (HGUMM), Murcia, Spain; 5 Department of Medicine, Centro Regional de Hemodonación, 30003, Murcia, Spain

**Keywords:** *PON1*, NQO1, EPHX1, Polymorphism, SNP, Lymphoma, Susceptibility

## Abstract

**Background:**

The interplay between genetic susceptibility and carcinogenic exposure is important in the development of haematopoietic malignancies. *EPHX1, NQO1* and *PON1* are three genes encoding proteins directly involved in the detoxification of potential carcinogens.

**Methods:**

We have studied the prevalence of three functional polymorphisms affecting these genes rs1051740 *EPHX1,* rs1800566 *NQO1* and rs662 *PON1* in 215 patients with lymphoma and 214 healthy controls.

**Results:**

Genotype frequencies for *EPHX* and *NQO1* polymorphisms did not show any correlation with disease. In contrast, the GG genotype in the *PON1* polymorphism was found to be strongly associated with the disease (15.3% vs. 4.7%; OR = 3.7 CI (95%): 1.8-7.7; p < 0.001). According to the pathological diagnosis this association was related to follicular (p = 0.004) and diffuse large B-cell (p = 0.016) lymphomas.

**Conclusions:**

Despite the fact that further confirmation is needed, this study shows that the *PON1* GG genotype in rs662 polymorphism could be a risk factor for B-cell lymphomas.

## Background

The development of lymphomas including Hodgkin’s (HL) and non-Hodgkin’s lymphoma (NHL) is, to a great extent, the result of a combined effect of genetic susceptibility and environmental factors. Xenobiotic metabolizing enzymes, such as EPHX1, NQO1 and PON1, play a role in the detoxification of potential carcinogens from chemical industries as well as endogenous compounds [1,2]. Although a considerable body of evidence has demonstrated that pesticide exposure may increase the risk of NHL [3,4], the association for chemical industry compounds as a risk factor for B-cell lymphoma remains controversial [5-7]. Polymorphisms in the genes coding these enzymes can be responsible for different susceptibilities to lymphoma development [8-10].

To our knowledge, few studies have analysed polymorphisms in genes coding for xenobiotic metabolism enzymes (such as EPHX1, NQO1 and PON1) in lymphoma [11] or leukemia [12,13]. The aim of our work was to study the genotype distribution of three missense single nucleotide polymorphisms (SNPs) (rs1051740, rs1800566 and rs662, respectively) in these genes in lymphoma patients and in healthy controls.

## Methods

### Study subjects

Blood samples were obtained from 215 lymphoma Caucasian patients −194 NHL and 21 HL residents of either Murcia or Cartagena, in the Southeast of Spain. Lymphoma classification and diagnose was based in the 2008 World Health Organization classification of lymphoma [14]. Our study subjects come largely from a previous described series of patients diagnosed between 2004 and 2010 at the HUSMR in Cartagena (n = 116) and Morales Meseguer University Hospital (HUMM) (n = 42) in Murcia [15]. The rest of participants included in the study cases (n = 57) were those diagnosed during 2011 who accepted participation in the study. All patients from the HUMM institution were follicular lymphoma (FL) and, for this reason, most of the patients included in this study (135/215) belonged to this type of lymphoma on the basis of the availability of DNA specimens from a previous study. Data on gender, age and place of residence were obtained from medical records. The control group comprised 214 unrelated Caucasian healthy blood donors matched for age, gender and geographical location without a previous history of malignancy, as previously described [15]. Informed consent was obtained from all subjects. The research was carried out in compliance with the Helsinki Declaration and the study was approved by the local Ethics Committee of both HUSMR and HUMM participating hospitals.

### Geographical study area

The geographical area under study included a specific zone (Escombreras’ Valley) with many chemical plants comprising one of the highest densities of heavy chemical industries in Spain [16-18] including an oil refinery, an electricity generating station using oil combustion, a combined cycle gas turbine, and a manufacturing plant for production of nitrogen and phosphoric fertilizers, as well as a biodiesel factory and a zinc and sulphuric acid extraction plant. The valley and its surrounding areas were therefore declared a “polluted atmospheric zone” by the Spanish government in 1979 and are therefore subjected to rigorous control and monitoring of atmospheric contaminants [18]. In fact, nitrogen oxides (NO_x_) as NO_2_, sulfurdioxide (SO2), chlorine (ClH) volatile organic compounds (VOCs) as benzene and mercaptans and particulate matter smaller than 10 microns (PM_10_) were amongst the most common pollutants in that area. Previous studies have confirmed a higher cancer incidence in this area compared with both the national average and the neighbouring municipalities [19,20].

### DNA extraction and genotyping

Total genomic DNA was obtained from blood samples using the automatic DNA extraction system Maxwell 16 and DNA extraction kit for blood samples (cat: AS1010) (Promega, Madison, USA) according to the manufacturer’s instructions. DNA was quantified by UV absorbance using the Biophotometer by Eppendorf (Hilden, Germany).

*NQO1* and *PON1* polymorphisms were determined by allelic discrimination using TaqMan probes and a 7500 F real-time PCR thermocycler both provided by Applied Biosystems (Foster City, CA). For the *EPHX* polymorphism we used the amplifluor SNPs genotyping system (Millipore, Darmstadt, Germany).

### Statistical analysis

Sample size reached in our series was optimum according to the sample size estimation for a gene-only study obtained with Quanto v.1.2 program (University of Southern California) [21]. Statistical analysis was performed using the SPSS computer program Version 15.0 (Chicago, Illinois, USA). Student’s *t*-test was used to compare mean age of cases and controls and Yeats-corrected Pearson *χ*^2^ test was used to evaluate statistical significance of genotype distribution in cases and controls. Given that xenobiotic metabolizing enzymes would behave as possible tumor suppressor by eliminating potential carcinogens, a recessive model for the p-value calculation was preferred, however the main results were also studied following dominant and co-dominant models. Hardy-Weinberg, linkage disequilibrium (LD) and joint effect analyses of the studied polymorphisms were performed using the online program Shesis Page ( http://analysis2.bio-x.cn/myAnalysis.php) [22].

## Results

The main demographical features of patients and controls are shown in Table 1. No significant differences were observed between patients and controls in terms of age and gender.

**Table 1:** Demographic features of cases and controls

	**n**	**Age (SD)**	**Female** **n (%)**
**Cases**	215	53.8 (15.2)	102 (47.4)
**Controls**	214	53.9 (8.4)	114 (53.3)
**p**		0.933	0.227
**total**	429	53.8 (12.8)	216 (50.3)

Genotyping was successfully performed on all study subjects except for nine cases which could not be genotyped for *EPHX* and two for *NQO1*. The genotype frequencies in the study population were EPHX1: TT 54.8%, TC 36.7%, CC 8.5%; NQO1: CC 59.5%, CT 35.0%, TT 5.4% and PON1: AA 42.7%, AG 47.3%, GG 10.0%, consistent with the Hardy-Weinberg equilibrium (p = 0.187, 0.892 and 0.228, respectively) and similar to the HapMap-CEU (European) frequencies (EPHX1: TT 46.4%, TC 41.1%, CC 12.5%; NQO1: CC 60.0%, CT 36.7%, TT 5.8% and PON1: AA 43.4%, AG 46.9%, GG 9.7%; [ http://hapmap.ncbi.nlm.nih.gov].

No significant differences were observed for the rs1800566 and rs1051740 polymorphisms in the *NQO1* and *EPHX*, respectively. In contrast, the GG genotype in the rs662 PON1 polymorphism was associated with lymphoma development (15.3% vs. 4.7%; OR = 3.7 CI (95%): 1.8-7.7; p < 0.001) (Table 2). The differences between cases and controls were also significant when considering allelic distributions (G: 165 (38.4%) vs. 124 (29.0%), respectively).

**Table 2:** ***EPHX1 *(rs1051740), *NQO1 *(rs1800566) and *PON1 *(rs662) genotype distributions according to gender**

		***EPHX1 *(rs1051740)**			***NQO1 *(rs1800566)**			***PON1 *(rs662)**		
		**TT**	**CT**	**CC**	**CC**	**CT**	**TT**	**AA**	**AG**	**GG**
**Total**	**Cases**	118 (57.3)	71 (34.5)	17 (8.2)	131 (61.5)	72 (33.8)	10 (4.7)	83 (38.6)	99 (46.0)	33 (15.3)
	**Controls**	113 (52.8)	83 (38.8)	18 (8.4)	123 (57.5)	78 (36.4)	13 (6.0)	100 (46.7)	104 (48.6)	10 (4.7)
	**p (OR; 95% CI)**	0.953 (1.0;0.5-2.0)			0.677 (0.8;0.3-1.8)			**0.0004 (3.7;1.8-7.7)**		
**Female**	**Cases**	57 (58.2)	36 (36.7)	5 (5.1)	63 (61.2)	34 (33.0)	6 (5.8)	42 (41.2)	47 (46.1)	13 (12.7)
	**Controls**	61 (53.5)	44 (38.6)	9 (7.9)	64 (56.1)	42 (36.8)	8 (7.0)	59 (51.8)	50 (43.9)	5 (4.4)
	**p (OR; 95% CI)**	0.590 (0.6;0.2-1.9)			0.936 (0.8;0.3-2.4)			0.05 (3.2;1.1-9.3)		
**Male**	**Cases**	61 (56.5)	34 (31.5)	13 (12.0)	68 (61.8)	38 (35.4)	4 (3.6)	41 (36.6)	52 (46.0)	20 (17.7)
	**Controls**	52 (52.0)	39 (39.0)	9 (9.0)	59 (59.0)	36 (36.0)	5 (5.0)	41 (41.0)	54 (54.0)	5 (5.0)
	**p (OR; 95% CI)**	0.627 (1.4;0.6-3.4)			0.884 (0.7;0.2-2.7)			**0.008 (4.1;1.5-11.3)**		

NA: not applicable.

The p-value calculated using the recessive model.

The association of rs662 with lymphoma was also obtained when applying the co-dominant model in the whole study population (p = 0.0009). When restricting for males and females p values were 0.016 and 0.054, respectively. However, no significant differences were obtained when the dominant model was used for any of the polymorphisms (data not shown).

As shown in Table 3, FL and diffuse large B-cell (DLBCL) lymphoma cases were more frequently GG carriers than controls. In contrast, HL cases did not show this association.

**Table 3:** ***EPHX1 *(rs1051740), *NQO1 *(rs1800566) and *PON1 *(rs662) genotype distributions according to the lymphoma diagnose**

	***EPHX1 (rs1051740)***			***NQO1 *(rs1800566)**			***PON1 (rs662)***		
	** CC**	** CT**	** TT**	** CC**	** CT**	** TT**	** AA**	** AG**	** GG**
**Controls**	113 (52.8)	83 (38.8)	18 (8.4)	123 (57.5)	78 (36.4)	13 (6.0)	100 (46.7)	104 (48.6)	10 (4.7)
**NHL**	109 (58.6)	65 (34.9)	12 (6.5)	122 (63.2)	63 (32.6)	8 (4.1)	76 (39.2)	88 (45.4)	30 (15.5)
**p (OR; 95% CI)**	0.458 (0.8;0.4-1.6)			0.513 (0.7;0.3-1.6)			**0.0005 (3.7;1.8-7.9)**		
**FL**	78 (59.5)	46 (35.1)	7 (5.3)	85 (63.0)	45 (33.3)	5 (3.7)	55 (40.7)	61 (45.2)	19 (14.1)
**p (OR; 95% CI)**	0.394 (0.6;0.2-1.5)			0.467 (0.6;0.2-1.7)			**0.004 (3.3;1.5-7.4)**		
**DLBCL**	21 (61.8)	10 (29.4)	3 (8.8)	19 (55.9)	12 (35.3)	3 (8.8)	11 (31.4)	18 (51.4)	6 (17.1)
**p (OR; 95% CI)**	0.8 (1.1;0.3-3.8)			0.818 (1.5;0.4-5.6)			**0.016 (4.2;1.4-12.5)**		
**HL**	9 (45.0)	9 (45.0)	2 (10.0)	9 (45.0)	9 (45.0)	2 (10.0)	7 (33.3)	11 (52.4)	3 (14.3)
**p (OR; 95% CI)**	0.861 (1.2;0.3-5.6)			0.835 (1.7;0.4-8.2)			0.181 (3.4; 0.9-13.5)		

NHL: Non-Hodgkin’s lymphoma. HL: Hodgkin’s lymphoma. FL: Follicular lymphoma. DLBCL: Diffuse large B-cell lymphoma. The p-value was calculated using the recessive model.

In order to ascertain the possible combined effect of the genotypes a joint effect analysis was performed and showed that the only significant combination was T-C-G for EPHX1, NQO1 and PON1 polymorphism, respectively rendering an OR of 1.73 (95% CI: 1.22-2.45; p = 0.002) (Table 4). As expected, D’ (<0.21) and r2 (<0.008) demonstrated that the polymorphisms studied were not in linkage disequilibrium.

**Table 4:** **Joint effect analysis of polymorphic loci for *EPHX1 *(rs1051740), *NQO1 *(rs1800566) and *PON1 *(rs662), respectively**

**Joint alleles**	**Case (freq)**	**Control (freq)**	**Fisher’s p**	**Pearson’s p**	**OR; 95% CI**
C C A	63.1 (0.16)	70.3 (0.16)	0.689	0.689	0.9; 0.6-1.3
C C G	22.3 (0.06)	21.8 (0.05)	0.836	0.836	0.9; 0.6-2.0
C T A	11.1 (0.03)	16.4 (0.04)	0.352	0.352	0.7; 0.3-1.5
T C A	136.2 (0.34)	166.3 (0.39)	0.078	0.078	0.8; 0.6-1.0
T C G	98.0 (0.24)	65.6 (0.15)	**0.002**	**0.002**	**1.7; 1.2-2.5**
T T A	43.2 (0.11)	51.0 (0.12)	0.511	0.511	0.9; 0.6-1.3
T T G	27.6 (0.07)	26.1 (0.06)	0.722	0.722	1.1; 0.6-1.9

All those frequency < 0.03 will be ignored in analysis.

## Discussion

Xenobiotic metabolising enzymes, such as EPHX1, NQO1 and PON1 play important roles in the detoxification of potential carcinogenic compounds. In fact, a plethora of studies have reported associations between polymorphisms in genes coding these enzymes and the risk of cancer development [8-10].

Epoxide hydrolase 1, encoded by*EPHX1* a gene located in 1q42.1, plays an important role in both the activation and detoxification of exogenous chemicals such as polycyclic aromatic hydrocarbons. Using *in vitro* expression studies Hassett et al. described that the substitution of His113 for the more commonly occurring Tyr113 residue in exon 3 decreased EPHX activity approximately 40% [23]. This single nucleotide polymorphism (SNP) (rs1051740) may play a role for genetic susceptibility to to childhood leukemia [24,25] and to lymphoma in males [10] whilst other studies have not found an association with susceptibility [26] and outcome [27] in leukemia. A recent study carried out the genotyping of 1,115 women (518 NHL and 597 controls) for six polymorphisms in genes involved in solvent metabolism (including EPHX1 rs1051740 and NQO1 rs1800566, but not PON1 rs662). In those women, different solvent exposures were measured, and some association was found between EPHX1 rs2234922 and DLBCL with a pattern of interaction with benzene exposure [11].

NAD(P)H dehydrogenase, quinone 1, a 2-electron reductase encoded by *NQO1* located in 16q22.1, detoxifies quinones derived from the oxidation of phenolic metabolites of benzene. The rs1800566 polymorphism consists of a C-to-T substitution at position 609, which codes for a Pro-to-Ser change at residue 187 (P187S). Exposition of wild type human bone marrow cells to hydroquinone (HQ) trigger an increase of NQO1 protein and activity, not induced in TT carriers [28]. Moreover, individuals homozygous for the TT genotype have an increased risk of benzene hematotoxicity and secondary cancers including leukaemia [29]. Some authors did find an association between this polymorphism and leukemia susceptibility [25,30,31] and outcome [27,32] whereas other studies, that included a meta-analysis, did not observed such an association [33-35].

Paraoxonase 1, encoded by *PON1* a gene located in 7q21.3, hydrolyzes the toxic oxon metabolites of several organophosphorous insecticides, as well as nerve agents, aromatic esters and a variety of aromatic and aliphatic lactones [36]. The *PON1* rs662 polymorphism consists of an A-to-G substitution that causes a Gln-to-Arg change at residue 192 (Q192R). Humbert et al. found that the allele encoding arginine has high-activity plasma paraoxonase, whereas glutamine at this position specifies a low-activity variant [37]. The role of this SNP in haematological malignancies has only been studied in two independent studies which found an association of rs662 with lymphohaematopoietic cancers [38] and lymphoma [9].

In our work, we studied the functional germline polymorphisms rs1051740, rs1800566 and rs662 in lymphoma patients and controls. Although the study subjects come from the same geographical area, important aspects concerning the source of cases and controls have to be taken into account. First, the controls do not represent exactly the general population since they are blood donors and second there is a degree of heterogeneity in the cases since they comprise several types of lymphoma that may be caused by different etiological factors. We found that rs1051740 or rs1800566 in EPHX1 and NQO1 were not related with lymphoma susceptibility as previously reported by others [8]. In contrast, we observed that rs662 in *PON1* was associated with lymphoma risk, being the GG genotype related with increased susceptibility to lymphoma in general, and to FL and DLBCL in particular. This finding is concordant with that reported by Kerridge et al. analysing DNA extracted from archival tumour specimens from a population of 169 NHL cases and 205 controls [9]. The rationale for performing the joint effect analysis of the studied SNPs is that, under a mixture of different pollutants, the contribution of polymorphisms in different metabolizing enzymes could exert an additive effect in disease susceptibility than can only be identified considering the SNPs as in combination. This analysis revealed that the combination of the PON1, EPHX1 and NQO1 polymorphisms did not increase the lymphoma risk associated with the PON1 polymorphism alone (OR = 1.7 vs. 1.5). The elegant study carried out by De Roos et al. in 1,172 cases and 982 controls found that another PON1 SNP (rs854560), but not rs662, was associated with a slightly increase of NHL [8]. Possible reason for this difference could be that the geographical characteristics pertaining to proximity to chemical industries were not the same or not considered. Taken together, our results could serve as a starting point for future studies in which both pollutant activity and the genotype influence could be studied. The identification of the type of compound or industry possibly implicated in this effect could be very interesting, but it is beyond the scope of this study. Recent genome-wide association studies (GWAS) have identified polymorphisms associated with lymphoma risk such as rs10484561 [39], rs2647012 [40] and rs6457327 [41] in the human leukocyte antigen (HLA) region on 6p21.32 and 6p21.33. Very recently, a novel region on 11q12.1 showed also association with lymphoma susceptibility [42]. Interestingly, none of these polymorphisms seems to lie on xenobiotic metabolizing genes. At this point, it is important to take into account that lymphomagenesis is a multifactorial process, and genetic-determined suboptimal xenobiotic metabolizing machinery could partly explain not all lymphoma cases but some of them, which have developed under certain exposure conditions. Geographical restriction of cases and controls is not a common feature of GWAS and, despite their undoubted utility in assessing the genetic determinants of the diseases, this approach could hamper the understanding of the contribution of xenobiotic exposure to lymphomagenesis.

## Conclusions

This is the first study demonstrating a relationship between the germline rs662 *PON1* polymorphism and lymphoma risk although this finding should be confirmed in larger and independent series.

## Abbreviations

DLBCL: Diffuse large B-cell lymphoma; EPHX1: Epoxide hydrolase 1; FL: Follicular lymphoma; HL: Hodgkin’s lymphoma; HQ: Hydroquinone; NHL: Non-Hodgkin’s lymphoma; LD: Linkage Disequilibrium; NQO1: NAD(P)H dehydrogenase, quinone 1; PCR: Polymerase chain reaction; PON1: Paraoxonase 1; SNP: Single nucleotide polymorphisms.

## Competing interests

The authors declare that they have no competing interests.

## Authors’ contributions

PCZ: Conception and design of the study, DNA extraction and manuscript drafting; JRC and DTM: Genetic analysis and interpretation of data; IE, MDGM and EPC: Analysis and interpretation of clinical data, JTS: Statistical analysis; RGC: DNA extraction and data analysis, JC, VV and MPG: Critical revision of the manuscript for important intellectual content. All authors read and approved the final manuscript.

## Pre-publication history

The pre-publication history for this paper can be accessed here:

http://www.biomedcentral.com/1471-2407/13/228/prepub
